# Supplementary figures and images for: The transmembrane protein Syndecan is required for stem cell survival and maintenance of their nuclear properties
Source: PLoS Genet. 2025 Feb 6;21(2):e1011586. doi: 10.1371/journal.pgen.1011586 (PMC11819509; doi:10.1371/journal.pgen.1011586)

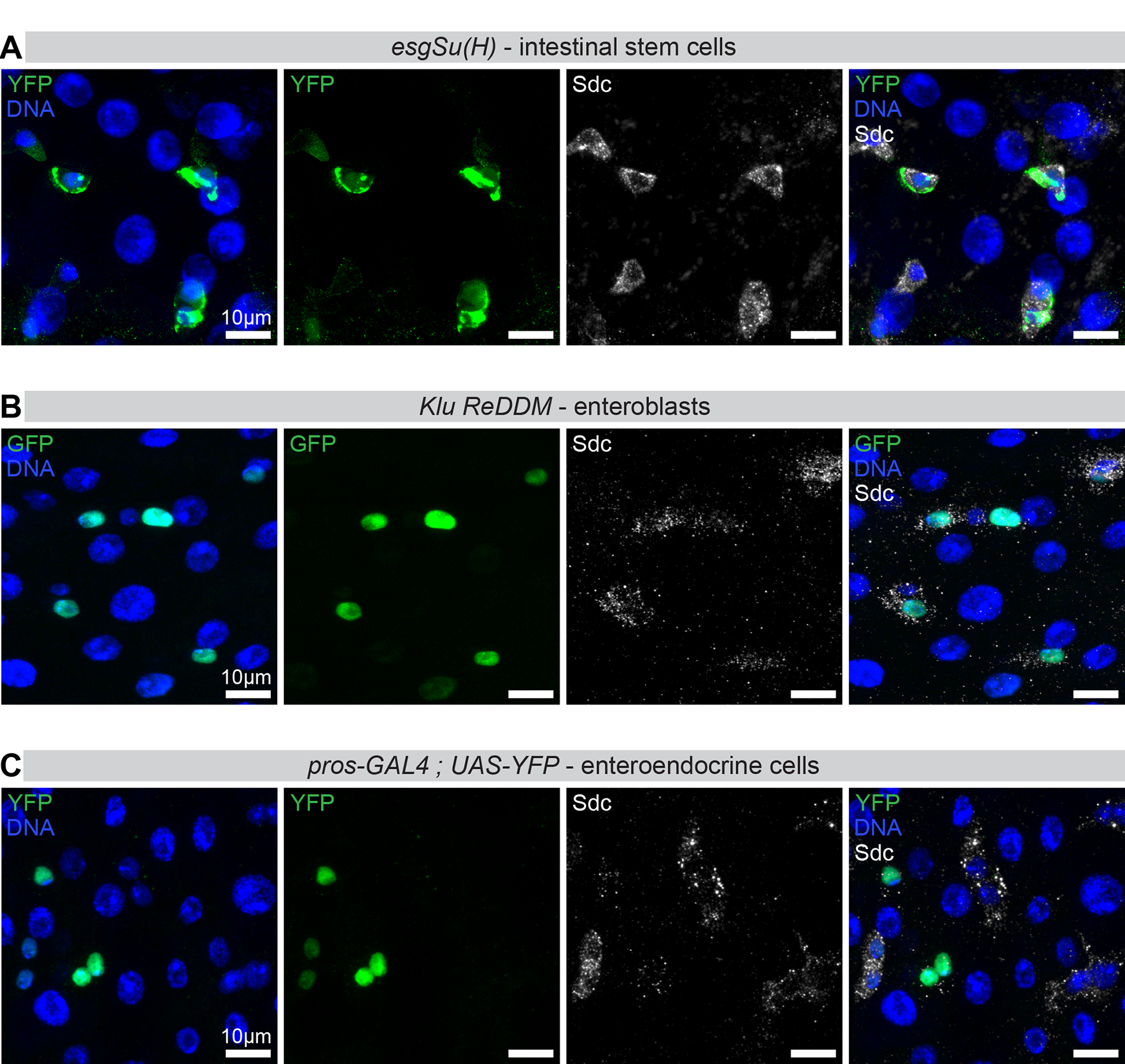

Supplement: S1 Fig — (A-C) Surface views of the R4/5 posterior midgut intestinal epithelium of flies carrying cell specific markers as indicated for each panel. Images are z projections through the intestinal epithelial depth, excluding the visceral muscle. DNA stain (blue) marks nuclei, anti-Sdc (white) marks Sdc protein. (TIF) [file pgen.1011586.s001.tif]

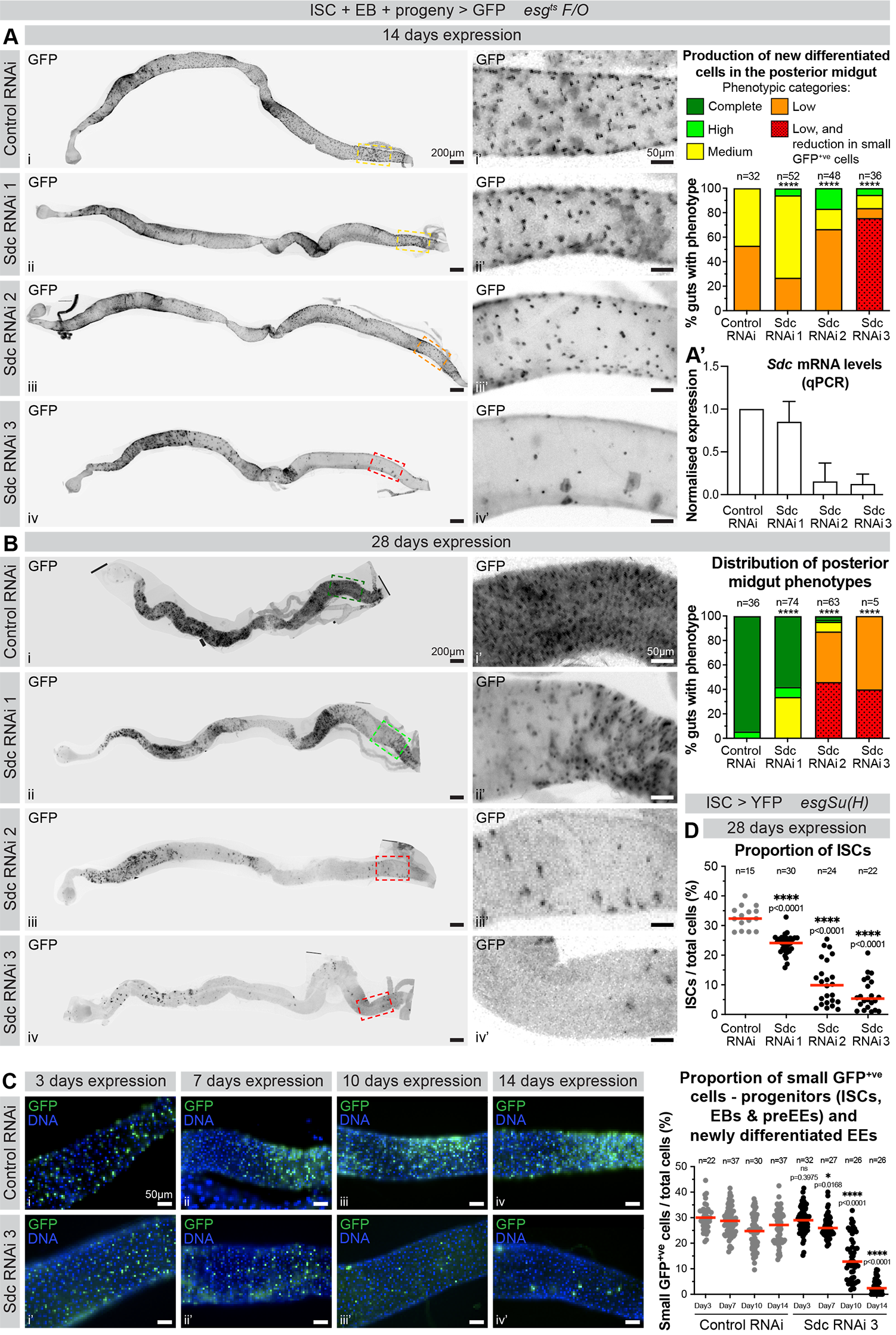

Supplement: S2 Fig — (A & B) Whole midgut views (i-iv) and posterior midgut zooms (i’-iv’) from flies expressing control RNAi (i) or one of three Sdc RNAi lines (ii-iv) using the esgts F/O system. Anterior left, posterior right. GFP (black) marks progenitor cells and their progeny. Dashed boxes indicate zoomed area, with the colour of the box indicating the phenotypic category to which the gut was assigned. Graphs show the distribution of posterior midgut phenotypes, with the size of the coloured bar representing the proportion of guts assigned to the phenotypic category in each genotype. n = number of guts, from three replicates. Fisher exact test, for all p < 0.0001, however in (A) Sdc RNAi 1 shows significance in the opposite phenotypic direction to Sdc RNAi 2 & 3. (A’) Quantification of sdc mRNA levels by qPCR (5 third instar larvae per biological replicate, n = 3 biological replicates per condition). Sdc RNAi lines were expressed with the ubiquitous tub-GAL4, UAS-CD8-GFP driver. (C) Surface views of midguts expressing control RNAi (i-iv) or Sdc RNAi 3 (i’-iv’) using the esgts F/O system. DNA stain (blue) marks nuclei; GFP (green) marks progenitor cells and their progeny. Graph shows the proportion of small GFP+ve cells (corresponding to progenitors (ISCs, EBs and pre-EEs) and newly differentiated EEs). n = number of guts, from three replicates. (D) ISC proportion in posterior midguts from flies expressing control or one of three Sdc RNAi lines for 28 days using the esgSu(H) system. n = number of guts, from three replicates. (TIF) [file pgen.1011586.s002.tif]

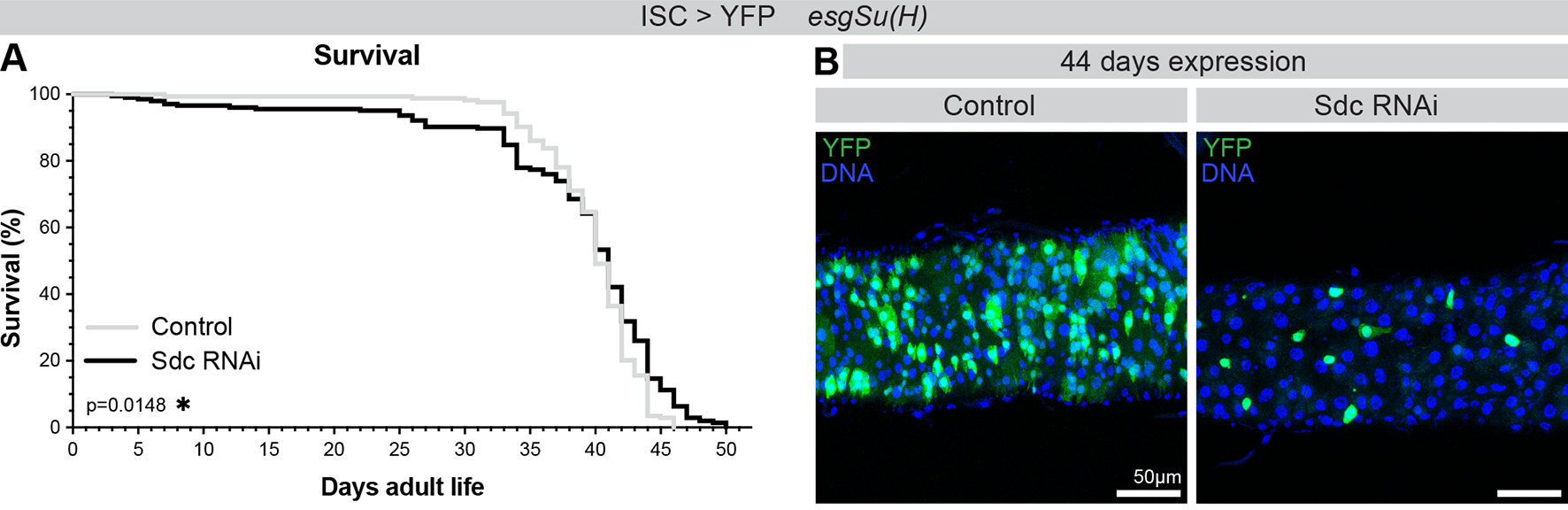

Supplement: S3 Fig — (A) Survival during continuous feeding (unchallenged conditions). Log-rank test. (B) Surface views of control midguts, or midguts expressing Sdc RNAi using the esgSu(H) system. DNA stain (blue) marks nuclei of all cells; YFP (green) marks ISCs. (TIF) [file pgen.1011586.s003.tif]

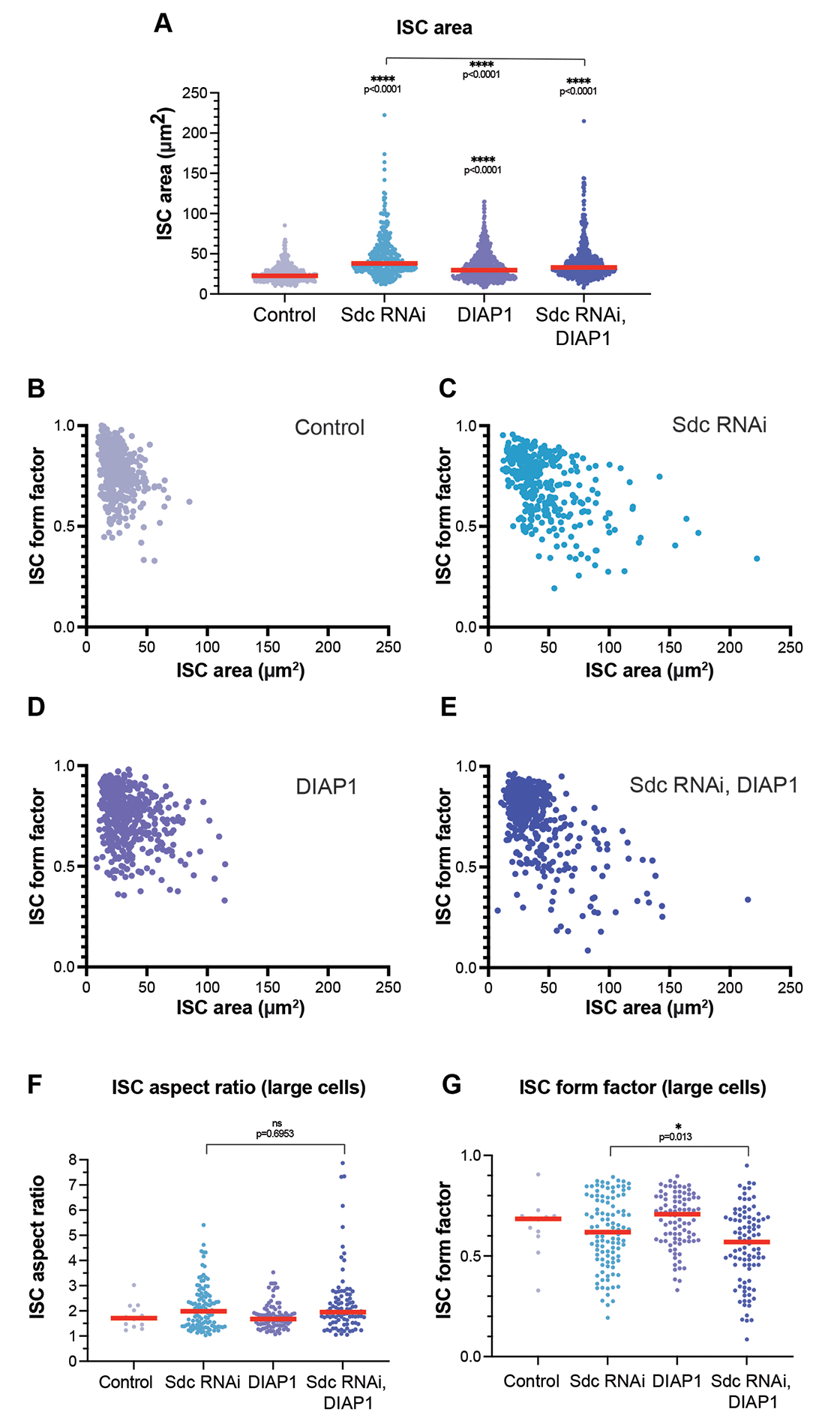

Supplement: S4 Fig — (A) ISC cytoplasmic area measured from YFP-expressing ISCs of indicated genotypes, across three biological replicates. (ISC numbers analysed: Control: n = 455, Sdc RNAi: n = 347, DIAP1: n = 530, Sdc RNAi, DIAP1: n = 480) (B-E) Correlation between ISC area and ISC form factor measured from YFP-expressing ISCs of indicated genotypes, across three biological replicates. (ISC numbers analysed: Control: n = 455, Sdc RNAi: n = 347, DIAP1: n = 530, Sdc RNAi, DIAP1: n = 480) (B) Control; (C) Sdc RNAi; (D) DIAP1; (E) Sdc RNAi, DIAP1. (F-G) ISC aspect ratio and ISC form factor shown for the subset of cells with an area>50μm2. (Subset of ISC analysed: Control: n = 12, Sdc RNAi: n = 111, DIAP1: n = 90, Sdc RNAi, DIAP1: n = 93). ISCs are larger and more convoluted upon Sdc depletion, and this is not suppressed by DIAP1 expression. (TIF) [file pgen.1011586.s004.tif]

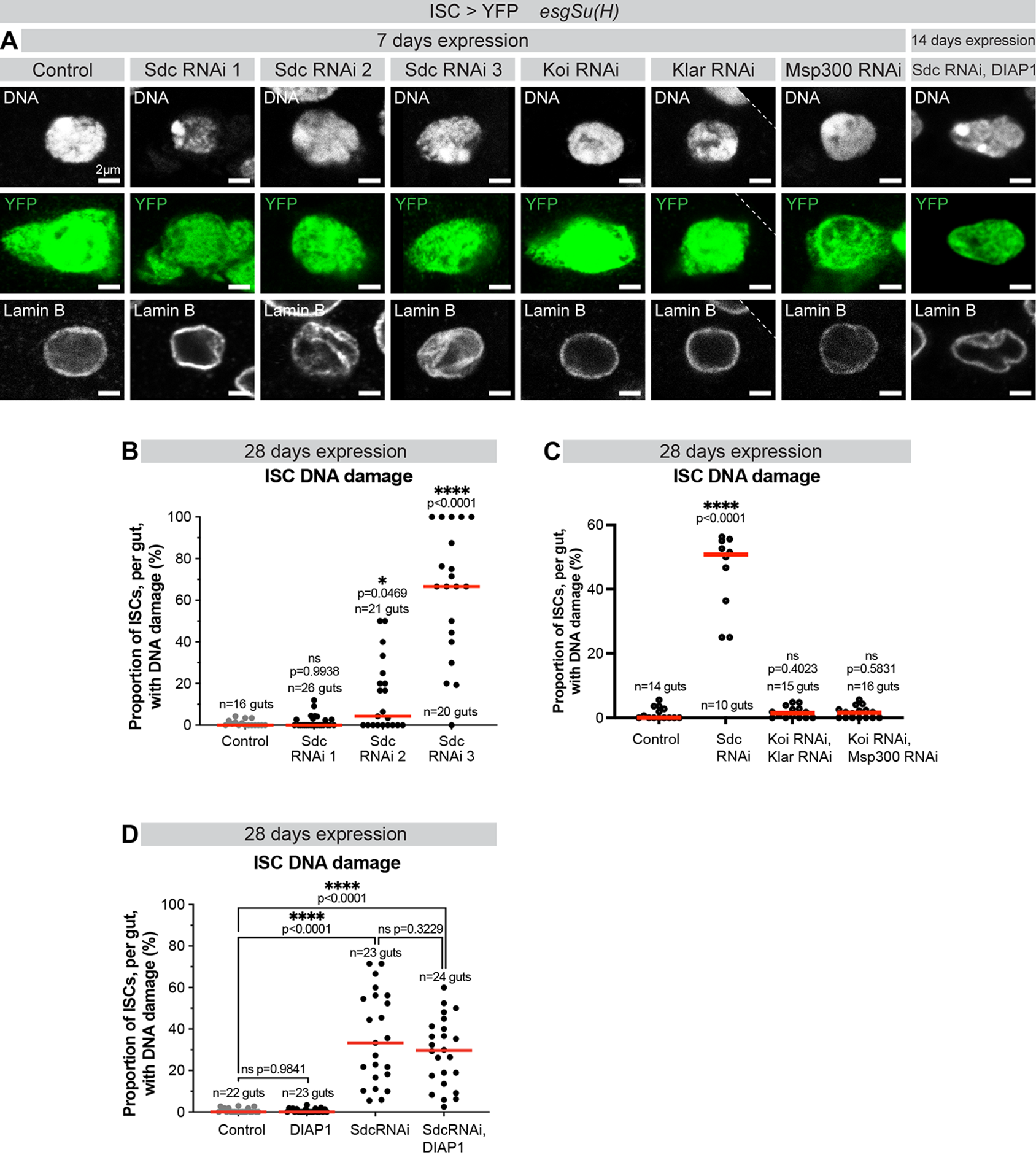

Supplement: S5 Fig — (A) Control ISC, and ISCs expressing various RNAis. DNA stain (white) marks nuclei; YFP (green) marks ISCs; anti-Lamin B (white) marks nuclear lamina. (B) Proportion of ISCs, per gut, with DNA damage. n = number of guts, from three replicates. >100 ISCs analysed per genotype. (C) Proportion of ISCs, per gut, with DNA damage. n = number of guts, from two replicates. >185 ISCs analysed per genotype. (D) Proportion of ISCs, per gut, with DNA damage. n = number of guts, from three replicates. >400 ISCs analysed per genotype. (TIF) [file pgen.1011586.s005.tif]

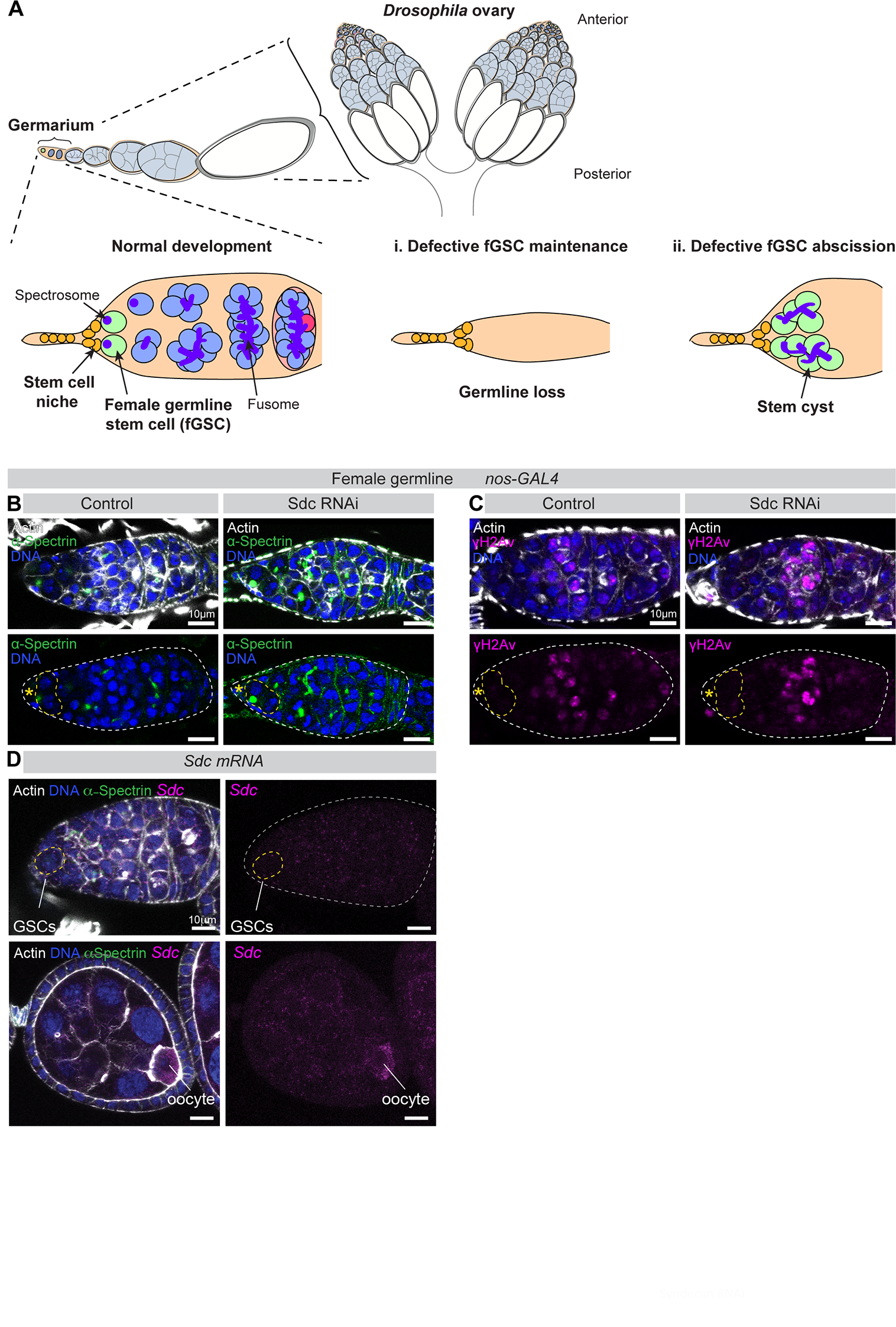

Supplement: S6 Fig — (A) Schematic of female germline development. Female germline stem cells (fGSCs) are maintained in a stem cell niche at the anterior of the ovary in a structure called the germarium. fGSCs provide an excellent model for identifying factors involved in stem cell maintenance (i) and abscission (ii), with clear phenotypic readouts [88]. (B) Germaria from control and germline-specific knockdown of Sdc. White dashed line outlines germarium, yellow dashed line outlines fGSCs, yellow asterisk indicates stem cell niche. DNA stain (blue) marks nuclei; Phalloidin (white) marks F-actin; anti-α-Spectrin (green) marks spectrosome/fusome, allowing identification of fGSCs. (C) Germaria from control and germline-specific knockdown of Sdc. White dashed line outlines germaria, yellow dashed line outlines fGSCs, yellow asterisk indicates stem cell niche. DNA stain (blue) marks nuclei; Phalloidin (white) marks F-actin; anti-γH2Av (magenta) marks DNA damage. (D) smFISH to label Sdc transcripts (magenta) in control germaria. Rest of tissue labelling as in B. Note the low Sdc expression in fGSCs. The bottom panels show higher Sdc expression at later stages, in the oocyte. (TIF) [file pgen.1011586.s006.tif]

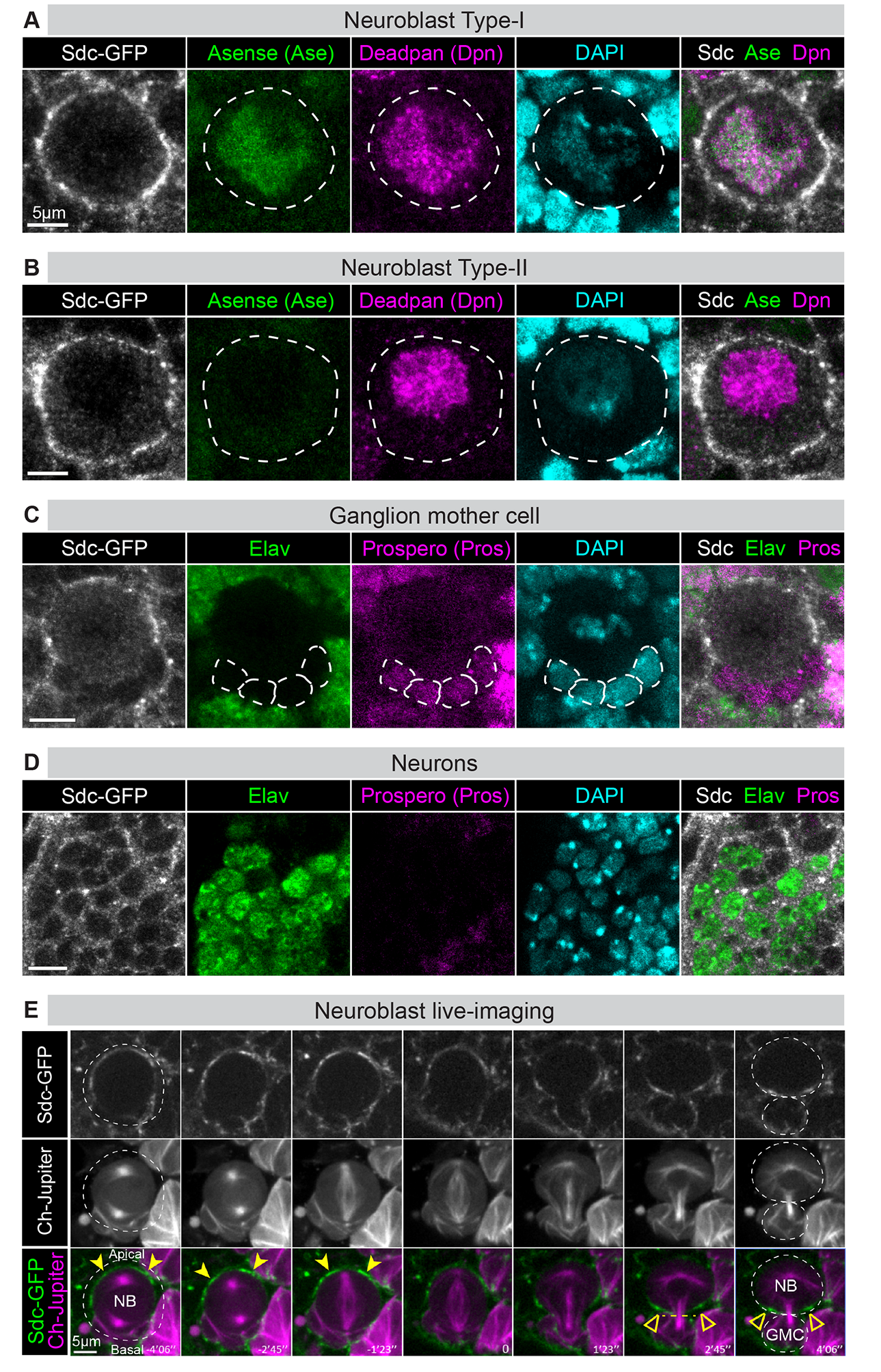

Supplement: S7 Fig — (A-D) Sdc::GFP-expressing larvae were dissected and immunostained for GFP and indicated markers to differentiate between type-I (Ase+ve Dpn+ve, the majority of neuroblasts) and type-II (Ase-ve Dpn+ve) neuroblasts, ganglion mother cells (Elav-ve Pros+ve) and neurons (Elav+ve Pros-ve). DAPI (cyan) marks DNA. Note the cortical Sdc distribution in neuroblasts. (E) Time lapse images of a mitotic neuroblast (NB, outlined with white dashed line) expressing Sdc::GFP (top row, and green in merge) and Cherry::Jupiter which marks the mitotic spindle (middle row, and magenta in merge). Prior to metaphase, Sdc::GFP localises to the apical cortex (filled yellow arrowheads). After anaphase onset, Sdc::GFP relocalises to the cleavage furrow (empty yellow arrowheads). Position of cleavage furrow indicated by yellow dashed line. In the final panel, NB labels the self-renewed neuroblast and GMC labels the forming daughter ganglion mother cell. Time stamp is in minutes and seconds, with anaphase defined as time 0. Images are single z sections. (TIF) [file pgen.1011586.s007.tif]

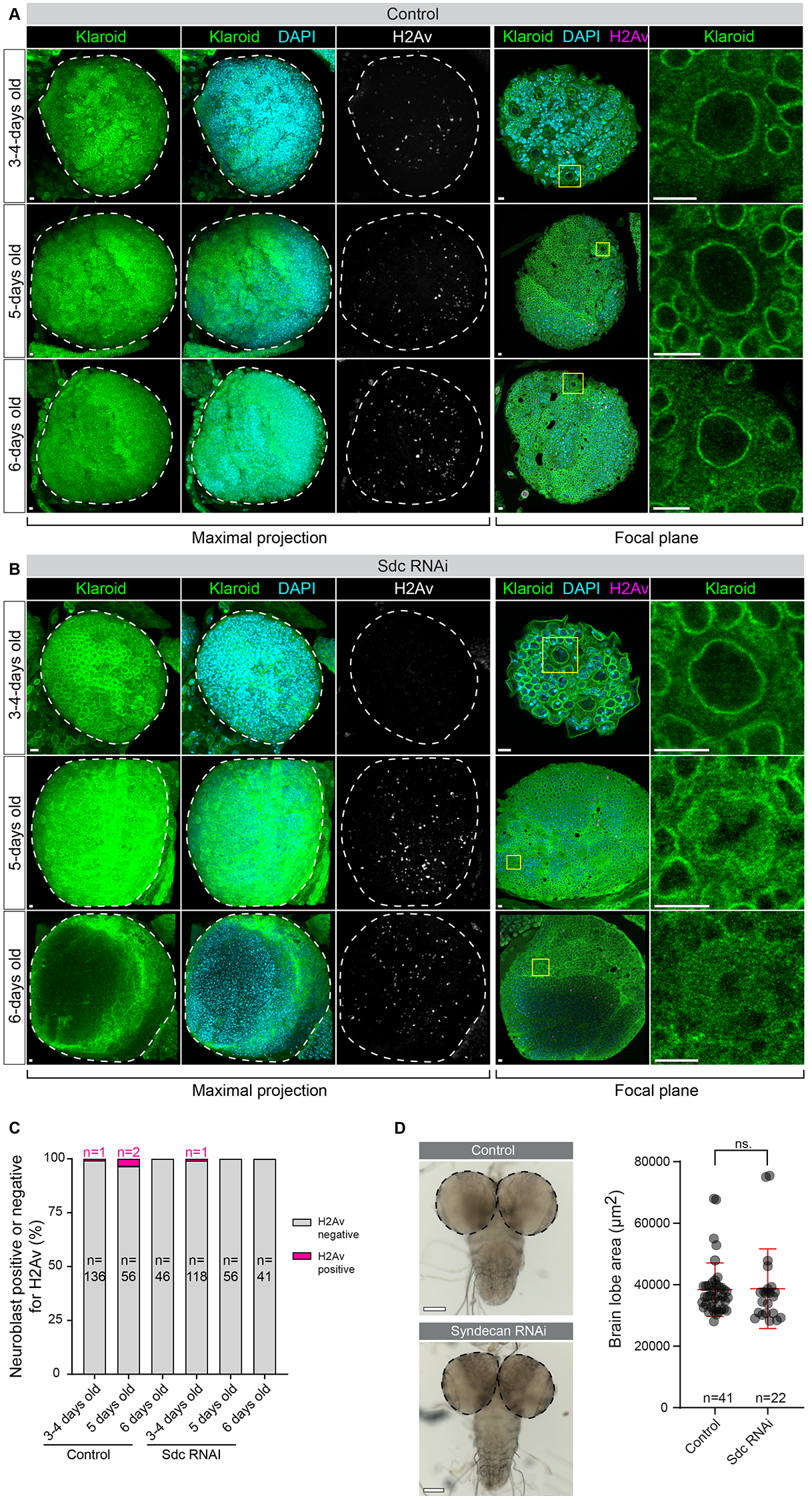

Supplement: S8 Fig — (A-B) Brain lobes labelled with Klaroid::GFP (nuclear envelope, green), DAPI (DNA, cyan) and anti-γH2Av (DNA damage, white/magenta). Brain lobes contain control (A) or Sdc-depleted neuroblasts (B). The larvae were dissected at indicated time points. Three panels on the left represent maximal projections of z-slices encompassing a whole brain lobe, two panels on the right show a single z-slice to better visualise the nuclear envelope. Note the nuclear envelope dispersion upon Sdc knockdown. Scale bars: 5 μm. (C) Quantification of the proportion of γH2Av-positive neuroblasts. Despite a general increase in anti- γH2Av signal as larvae age (see A, B), Sdc-depleted neuroblasts do not acquire DNA damage. (D) Representative pictures of dissected brains from 5-day to 5.5-day old larvae and associated quantitative measures across two independent experiments (n=number of brain lobes). Brain lobes are outlined with dotted lines. Scale bar: 100 μm. (TIF) [file pgen.1011586.s008.tif]

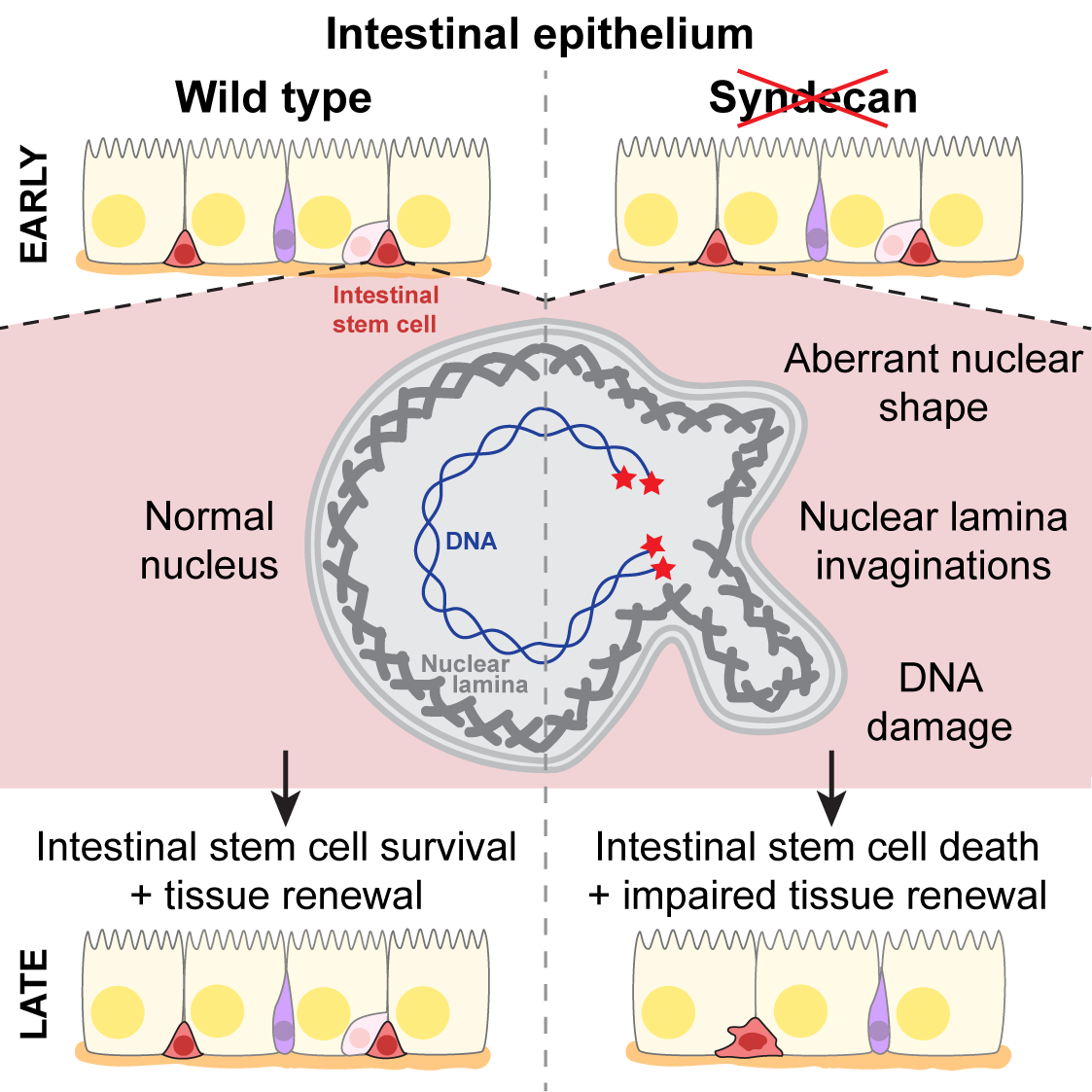

Supplement: S9 Fig — (TIF) [file pgen.1011586.s009.tif]
