# Supplementary material for: The transmembrane protein Syndecan is required for stem cell survival and maintenance of their nuclear properties
Source: PLoS Genet. 2025 Feb 6;21(2):e1011586. doi: 10.1371/journal.pgen.1011586 (PMC11819509; doi:10.1371/journal.pgen.1011586)
Supplement: Attachment — Submitted filename: 144437_1_rebuttal_3000881_sccmyw.docx [file pgen.1011586.s020.docx]

­­­­­

**Manuscript number:** RC-2024-02398

**Corresponding author(s):** Golnar Kolahgar

*[The “revision plan” should delineate the revisions that authors intend to carry out in response to the points raised by the referees. It also provides the authors with the opportunity to explain their view of the paper and of the referee reports.*

*The document is important for the editors of affiliate journals when they make a first decision on the transferred manuscript. It will also be useful to readers of the reprint and help them to obtain a balanced view of the paper.*

*If you wish to submit a full revision, please use our "*[*Full Revision*](https://reviewcommons.org/templates/full-revision.docx)*" template.* ***It is important to use the appropriate template to clearly inform the editors of your intentions.****]*

## 1. General Statements [optional]

*This section is optional. Insert here any general statements you wish to make about the goal of the study or about the reviews.*

With this study, we show for the first time a link between the transmembrane protein Syndecan and stem cell nuclear morphology and fate. We are pleased that the reviewers broadly agree that this information opens many interesting and important questions about the mechanistic links between Syndecan, the nucleus and stem cell behavior, which will be relevant to cell, stem cell and cancer biologists. We are grateful for their assessment of our work and constructive suggestions to improve the manuscript. In response to their comments, we suggest revising the manuscript to incorporate additional information and/or correct some statements.

## 2. Description of the planned revisions

*Insert here a point-by-point reply that explains what revisions, additional experimentations and analyses are planned to address the points raised by the referees.*

Below, we describe our plan to address most of the reviewers’ queries.

**Reviewer #1 (**Evidence, reproducibility and clarity (Required)):

**Summary:** In this paper the authors explore the function of Syndecan in Drosophila stem cells focussing primarily on the intestinal stem cells. They use RNAi knockdown to conclude that Syndecan is required for long term stem cell maintenance as its knockdown results in apoptosis. They suggest that this effect is independent of LINC complex proteins but is associated with changes to nuclear morphology and DNA damage. They go on to show that a similar impact on nuclear shape can be seen in larval neuroblasts but not in stem cells of the female germline.

**Major Comments:** The key conclusion that underpins the paper is that reduced Syndecan causes loss of stem cells. This is based entirely on evidence from cell-type specific RNAi using 3 independent RNAi lines. Overexpression has no phenotype and there is no analysis of loss of function mutants. SdcRNAi3 gives strong phenotypes that are statistically significant and is used throughout the paper. SdcRNAi2 gives comparatively moderate phenotypes which trend in the same direction but it is not clear if these are statistically significant (Fig S1). SdcRNAi line 1 appears to have very little effect (and if anything trends in the opposite direction in S1A). In addition, the knockdown efficiency of the three lines has not been assessed. Another possible concern given the dependence on RNAi3 is that the RNAi control line used is not an ideal match for the VDRC GD RNAi lines as it is in a different genetic background. In order to robustly draw conclusions: the phenotypes with RNAi lines 1 and 2 should be tested for significance; the extent of knockdown in each should be quantified either by qPCR in whole tissue knockdown, or by staining for protein levels if possible, to assess whether the variation in phenotypes is due to different knockdown levels. The use of a loss of function mutant in clones or tissue specific CRISPR-Cas9 KO or KD would also significantly increase confidence in the findings.

- Our qPCR data indicate that SdcRNAi3 produces the most efficient knockdown, whilst SdcRNAi1 generates the weakest knockdown. The new manuscript version will incorporate this data in figure S1. Knockdown efficacy of SdcRNAi 3 has also been previously reported (Eveland et al., 2016).

- We apologise for omitting to add the statistical tests on phenotypic categories in figure S1A, this will be revised. We confirm that all Sdc RNAi phenotypic distributions are significantly different to that seen for age-matched controls (p<0.0001 for all).

- It should also be noted that despite weaker knockdowns with SdcRNAi1 and 2, we still observed statistically significant ISC depletion after 28 days of RNAi expression - we will add this data in figure S1. Overall, we are confident about Sdc’s role in maintaining intestinal stem cells.

Similarly, the evidence for a lack of LINC protein role in the phenotype relies on single RNAi lines without validation of knockdowns. The authors should ideally validate these lines in this system or reference other studies that have validated the lines in this or other contexts.

- The klarsicht RNAi line (BDSC 36721) and klaroid RNAi line (BDSC 40924) used in this study have been validated and used in other studies. (Falo-Sanjuan & Bray, 2022; Collins et al., 2017)

- For Msp300 RNAi knockdown we have used two independent RNAi lines which gave similar results. We will amend the text to clarify these points. In addition, the line reported in the manuscript was previously validated (Dondi et al., 2021; Frost et al., 2016).

**Minor Comments:** The figures are generally very clear but some of the IF image panels are very small and require significant on-screen enlargement to be legible. In particular in Figure 1B the cross section views make it difficult to assess expression in the different cell types (and don't show very many cells), could this be shown in wholemount or as separated channels in a supplementary figure? In addition, it would strengthen the argument to include counterstains for markers of the different cell types (particularly to distinguish ISC/EB from EE). This could include esg-lacZ to mark ISC/EBs or prospero for EEs. However, if a broader view of these panels makes it clearer that all epithelial cells are expressing Syndecan this may not be essential.

- We are happy to incorporate larger fields of view, and co-immunostaining with different cell type markers.

Syndecan is referred to throughout as a stem cell regulator. This implies that in certain contexts or in response to certain stimuli its expression may be altered to elicit a stem cell response but no examples of this are shown. Moreover, only knockdown and not overexpression gives phenotypes suggesting its role may be as a required protein than a regulator. Either examples of its expression being modulated in homeostasis or in response to a challenge could be included or the wording could be amended.

- We agree with the reviewer and will amend the wording.

Expression of Syndecan in neuroblasts is described as data not shown, it would be better to include this for completeness.

- We will add this data in figure 4.

In addition to the intestinal validation of the Syndecan RNAi lines, validation of knockdown in the germline would be valuable to support the conclusions of Fig S4 given differences of knockdown in the germline with some RNAi lines (although inclusion of Dicer in the driver line should have overcome this).

- Sdc expression is very low in the germline, compared to the surrounding somatic cells, therefore we are not confident that we can detect differences in expression level after knockdown. We suggest adding a panel in figure S4 to show the low expression and adding a comment in the text.

**Reviewer #1 (Significance (Required)):** The study describes a potentially very interesting, novel link between Syndecan, nuclear shape and apoptosis in cycling cells that could have broad relevance. If fully validated this could have implications for other stem cell populations, including those in mammals and disease relevance in the context of cancer. The paper is fundamentally descriptive in nature and so the level of significance hinges on the strength of evidence and how interesting the phenotype itself is. At this stage the audience will be primarily in the areas of fundamental research in biology of the nucleus and cytoskeleton. Defining the mechanistic link between Syndecan and nuclear morphology will be a critical next step and while not essential for this study would significantly increase the likely interest in the paper.

- We thank the reviewer for these constructive comments. We agree that discovering the mechanistic links between Syndecan and nuclear morphology in future studies, in this and other model systems, will be relevant to many areas of biological research.

In terms of significance in stem cell biology the distinction between a regulator and a requirement to prevent stem cell apoptosis is important and the lack of evidence for a context in which Syndecan plays a regulatory role somewhat detracts from the breadth of impact. My field of expertise is in epithelial stem cell biology.

- We agree and will amend our wording.

**Reviewer #2** (Evidence, reproducibility and clarity (Required)): 
**Summary:** Stem cell (SC) maintenance and proliferation are necessary for tissue morphogenesis and homeostasis. The basement membrane (BM) has been shown to play a key role in regulating stem cell behavior. In this work, the authors unravel a new connection between the receptor for BM components Syndecan (Sdc) and SC behavior, using Drosophila as model system. They show that Sdc is required for intestine stem cell (ISC) maintenance, as Sdc depletion results in their progressive loss. At a cellular level, they also find that Sdc depletion in ISCs affects cell survival, cell and nuclear shape, nuclear lamina and DNA damage. In addition, they show that the defects in shape are not related to cell death. They also find that Sdc depletion in neural stem cells also results in nuclear envelope remodeling during cell division. This is in contrast to what happens in female germline stem cells where Sdc does not seem to be required for their survival or maintenance. 
In general, I believe that this work unravels a connection between Sdc and stem cell behavior. However, I think the study is still at a preliminary stage, as how Sdc regulates different facets of stem cell behavior remains unclear. 

**Major comments:** 
1. To clearly show that the cellular changes produced by loss of Sdc are not due to cell death, one should quantify the ISC area and shape of Sdc-depleted ISCs expressing DIAP1 and compare it to that of Sdc-depleted ISCs. As DIAP1 overexpression only partially rescues ISC loss due to Sdc depletion, one should show that the Sdc-depleted ISCs expressing DIAP1 that still show cellular changes are not dying, as overexpression of Diap1 might not be sufficient to completely rescue cell death in all Sdc-depleted ISCs. In fact, apoptosis in Sdc depleted guts and the ability of Diap1 overexpression to rescue cell death should be analyzed using markers of caspase activity, this will provide a better idea of the contribution of apoptosis to the phenotypes associated to Sdc depletion.

- We can, as suggested by the reviewer, quantify the area and shape of Sdc-depleted ISCs expressing DIAP1 and compare it to that of Sdc-depleted ISCs. However, our immunostainings with anti-Caspase 3 or Drice do not pick up apoptotic cells in the fly gut. This is not entirely unexpected, as apoptosis is unfortunately not easily detected in this tissue. In the absence of a positive readout of apoptosis, we will not be able to discriminate between apoptotic and non-apoptotic stem cells when quantifying area and shape and will only have global quantifications.

2. The authors show that ISC loss is associated with reduced cell density, suggesting that this is most likely due to failure in new cell production. What do they mean with cell production? Is this related to a problem in regulating cell division or to the fact that as some ISCs are lost by apoptosis there is progressively less ISCs or to a combination of both? I think that cell division should be monitored throughout time as well as cell death in ISCs.

- Based on esgF/O experiments (fig. 1D-F and S1C) where we can trace the production of new cells with GFP, we know that Sdc RNAi expression (i) impairs the appearance of newly differentiated cells in the tissue and (ii) results in the disappearance of progenitor cells (fig. S1C). Supporting these points, (i) we have observed PH3+ mitotic stem cells upon Sdc RNAi, so we are confident the cells are able to initiate cell division (see also fig. 2G), and (ii) we have occasionally noted in fixed samples stem cells looking like they were in the process of delaminating. Overall, the failure of cell production is likely related to problems with both completion of cell division and progressive stem cell loss. High resolution live imaging will in future give us a better insight into stem cell division dynamics/behaviour, however, the technical improvements required are beyond the scope of this project. In the meantime, we propose to clarify our statement in the text.

3. The authors report that in contrast to what happens when Sdc is eliminated from ISCs, its elimination from EEs results in an increase in the number of these cells. An explanation for this result is missing.

- Based on known roles of Syndecan in other *Drosophila* tissues (Johnson et al., 2004; Steigemann et al., 2004; Chanana et al., 2009; Schulz et al., 2011), we speculate that Syndecan may contribute to robo/slit signalling, which is an important regulator of EE activity in the *Drosophila* gut (Biteau & Jasper 2014; Zeng et al., 2015). We propose to amend the text to express this hypothesis.

4. The authors suggest that "Sdc function is unlikely to be fully accounted for by individual LINC complex proteins, although these proteins might act redundantly". Checking redundancy seems a straight forward experiment, which only requires the simultaneous expression of RNAis against several of these proteins. This would help to settle the implication of LINC complex proteins on Sdc function. 

- To check redundancy, we propose to combine Klaroid RNAi with Msp300 or Klarsicht RNAis, and express two RNAis at a time in ISCs. We will then measure stem cell proportions and the proportion of ISCs with DNA damage.

5. Although quantification of DNA damage, by immunolabelling with γH2Av, reveals that knockdown of individual LINC complex components did not recapitulate the damage observed upon Sdc depletion (Fig.3G), the image shown in Fig.3F reflects much higher levels of γH2Av in Msp300 RNAi cells compared to Sdc RNAi cells. Authors should clarify this.

- Like the reviewer, we are intrigued by the higher levels of H2Av staining in the tissue, despite Msp300 knockdown in stem cells only (fig. 3F). It is worth noting that we observed this with two independent RNAi lines (we showed only one RNAi in the manuscript, but we will amend the text to indicate this). In fig. 3F, we will indicate with an arrow the only ISC that is H2Av positive, and mention in the text that the majority of DNA damage signal observed in the Msp300 RNAi condition is in enterocytes, not ISCs. We currently do not have an explanation for why loss of Msp300 in ISCs should cause DNA damage in neighboring cells.

In addition, the consequences of the simultaneous elimination of more than one component of the LINC complex on DNA damage should be analyzed.

- We agree, and as we check for redundancy (as in point 4), we will also immunostain the tissues for H2Av.

6. The authors claim that the fact that "DNA damage was found more frequently in Sdc-depleted ISCs with lamina invaginations compared to those without (Figure 3H), supports a model whereby the development of nuclear lamina invaginations precedes the acquisition of DNA damage". However, to me, these results show that there is a relation between these two phenotypes, but not that one precedes the other. In order to show which one is the possible cause and which the consequence, the authors should perform a time course of the appearance of each of these phenotypes.

- We agree with the reviewer that we should rephrase our statement to indicate a relationship between lamina invaginations and DNA damage, rather than a causality (as stated in fig. 3H).

(In terms of performing a time course analysis, the difficulty is that after 3 days of Sdc RNAi expression, the apparent DNA damage (fig. 3G) corresponds to a very small proportion of stem cells, meaning that an exceptionally large sample size would be required to achieve robust statistical analysis.)

7. When studying the role of Sdc in neural stem cells, the authors show that elimination of Sdc in neuroblasts also affect nuclear envelope and shape. Furthermore, in this case, they also show that Sdc elimination affects cell division. To look for a more conserved role of Sdc in stem cell behavior, I believe the authors should also analyze whether Sdc elimination in neural stem cells results in an increase in DNA damage, as it is the case in ISCs.

- We will stain larval brains for H2Av to see if DNA damage is also observed following Sdc knockdown in neuroblasts.

8. When analyzing a possible role of Sdc in fGSCs, quantification of germline stem cells and γH2Av levels in control nosGal4 and nos>Sdc RNAi germaria should be done. In addition, it is not clear to me whether Sdc is in fact expressed in fGSCs.

- As mentioned in comments to reviewer 1, we will add a panel in figure S4 to show the low Sdc expression in fGSCs. We will also clarify in the text that we do not see any H2Av staining in the fGSCs (thus, there is nothing to quantify in this case).

9. The authors should show presence of Sdc in neuroblasts.

- Yes, we agree, as also mentioned in comments to reviewer 1.

**Reviewer #2 (Significance (Required)):** In general, although this work reveals that elimination of Sdc affects different aspects of intestinal and neural stem cell behavior, including cell survival, cell production, nuclear shape, nuclear lamina or DNA damage, their contribution to stem cell loss and interactions between them have not been analyzed in detail. The role of the basement membrane in stem cell behavior has been extensively studied. In particular, the role of syndecan in stem cell regulation has been primarily confined to cancer, muscle, neural and hematopoietic stem cells. Thus, the study here presented could extend the role of Sdc to intestinal stem cells and could potentially reveals a conserved role for Sdc in neural stem cell behavior. However, the problem with the data mentioned above, hinders the assessment of the significance of this work. 

- We thank the reviewer for their assessment and are glad that they also find that our study provides novel connections between Syndecan and the regulation of intestinal and neural stem cell behaviors. To strengthen our conclusions, we will include additional experiments or amend the text, as indicated above.

**Reviewer #3** (Evidence, reproducibility and clarity (Required)): 
Peer-review: The transmembrane protein Syndecan regulates stem cell nuclear properties and cell maintenance. 

In this work, the authors investigate the role of the transmembrane protein Syndecan (Sdc) in nuclear organisation and stem cell maintenance. Theys show that Sdc knockdown in intestinal stem cells (ISCs) results in a reduction of the ISC pool as well as of their progeny. They hypothesise that these ISCs might get eliminated via cell death, however, expression of the apoptotic inhibitor DIAP1 only rescued ISC loss by 50%. Hence, they suggest that apoptosis can not account for the total decrease in ISCs observed upon Sdc loss. 
ISCs depleted from Sdc exhibited abnormal cytoplasmic and nuclear morphologies. As Sdc has previously been implicated in the abscission machinery in mammalian cultured cells, they tested if Sdc could be playing a similar role in the abscission of ISCs. However, ISCs were capable of undergoing cytokinesis. Next, they tested if Sdc depletion could be altering the linkage between the plasma membrane and the nucleus mediated by the Linker of Nucleoskeleton and Cytoskeleton (LINC) complex. However, individual knockdowns of the different components of the complex did not disrupt the nuclear morphology to the same extent as Sdc knockdown, suggesting that Sdc function may be independent of the LINC complex. Finally, they observed that Sdc-depleted ISCs exhibited DNA damage, suggesting that Sdc may play a role in DNA protection. 
The authors next tested if Sdc played similar roles in other stem cell types such as the female germline stem cells (fGSCs) and larval neural stem cells (NSCs). While Sdc depletion appeared dispensable for fGSC maintenance, it prolonged NSC divisions and altered the nuclear morphology of NSCs. Upon further investigations, they observed that the NSC's nuclear envelope was disrupted upon division, hence causing defects in the nuclear size ratio of NSC and their progeny. 
This study provides with interesting findings in the field and proves a new role for Sdc in the regulation of intestinal and neural stem cell maintenance. I would recommend this manuscript to be accepted if the authors address the following comments. 

Major comments: 
1. In Figure 2 A-B, Sdc RNAi should ideally have a UAS control transgene to match the number of UAS being expressed to that of Sdc RNAi, DIAP1. Otherwise, it is plausible that reduced RNAi expression of Sdc RNAi, DIAP1 animals is the cause of the partial rescue. Staining against cell death markers such as Dcp-1 or TUNEL might also quantify the number of cells undergoing cell death in each of the genotypes.

- As mentioned in comments to reviewer 2 (point 1), it is difficult to label apoptotic cells in the fly gut. However, we could set up an additional control to test that the partial rescue observed upon DIAP1 expression is not a result of Gal4 dilution.

2. " These phenotypes were observed both with and without DIAP1 expression (Figure 2C), indicating that these cell shapes are not caused by apoptosis." Misleading, as DIAP overexpression in Sdc knockdown background only rescued apoptosis by 50%. Hence, it is possible that those cells undergoing morphological defects, protrusions and blebbing might still undergo death - also considering those morphological changes are typically observed in apoptotic cells... Therefore, to rule apoptosis out, these cells should be shown to be negative for cell death markers.

- We agree, however, it is difficult to label apoptotic cells. We think that the quantification of shape and area (as suggested by reviewer 2, point 1) will clearly show that the cell shapes resulting from Sdc depletion are not caused by apoptosis.

3. Show if Sdc is expressed in fGSCs - the lack of phenotype caused by Sdc knockdown might be due to lack of expression of Sdc.

- As mentioned in comments to reviewers 1&2, we will add a panel in figure S4 to show the low *sdc* expression in fGSCs.

4. "After confirming the presence of Sdc in neuroblasts (data not shown)." Data should be shown. It would be of great interest for researchers if you showed a staining of different brain cell types (NBs, glia, neurons) and the Sdc expression patterns.

- As mentioned in comments to reviewers 1&2, we will add a panel in figure 4 to show *sdc* expression in NBs and the overall expression pattern.

5. You show how Slc-depleted NBs have disrupted nuclear morphologies. However, does Slc KD in NB lineages affect their ability to self-renew and generate differentiated progeny? Is the number of NBs and of their progeny cells altered as it is for ISCs?

- We propose to knockdown Sdc in NBs and quantify brain size in 3^rd^ instar larvae to test if the ability to generate progeny is affected.

6. Does protection against DNA damage in an Slc knockdown background prevent the defects observed with the single knockdown and ISC elimination?

- This is a good question, and we should emphasize this point in the discussion. However, because of the multiple routes of DNA damage response, and the multiple lines needed to explore this connection, we feel that investigating this question is beyond this project.

7. Any idea the similarities between ISC and NBs that can account for why Sdc knockdown has effects in those systems, while no effect was observed in the germ cells?

- Besides the differences in expression level, we speculate that GSCs may have a different nuclear / lamina architecture which might reflect differences in how GSCs control the physical integrity of their nuclei. It is also possible that the differences observed between tissues reflect the way stem cells connect to their microenvironment. Notably, fGSCs rely extensively on E-Cadherin mediated adhesion with neighbouring cells, and it is possible that contact with the extracellular matrix is dispensable. We will consider these possibilities in the discussion.

**Minor comments:** 
8. Lamina invaginations, for example in Figure 3 A, could be indicated with an arrow for easier detection.

- Thanks for this suggestion, we will amend the figure.

9. Specify the type and location of NB imaged during live cell experiments.

- The NBs were imaged in the brain lobes, and we did not distinguish between type I and II NBs. We will add a sentence in the method section to clarify.

Reviewer #3 (Significance (Required)): 
Expertise: Drosophila stem cells

- Many thanks for the constructive comments.

## 3. Description of the revisions that have already been incorporated in the transferred manuscript

*Please insert a point-by-point reply describing the revisions that were already carried out and included in the transferred manuscript. If no revisions have been carried out yet, please leave this section empty.*

n/a

## 4. Description of analyses that authors prefer not to carry out

*Please include a point-by-point response explaining why some of the requested data or additional analyses might not be necessary or cannot be provided within the scope of a revision. This can be due to time or resource limitations or in case of disagreement about the necessity of such additional data given the scope of the study. Please leave empty if not applicable.*

As described in section 2, we are confident we can address most of the comments. We have indicated when we feel an experiment might be beyond the scope of a revision (reviewer 3, point 6).
