# Supplementary material for: The transmembrane protein Syndecan is required for stem cell survival and maintenance of their nuclear properties
Source: PLoS Genet. 2025 Feb 6;21(2):e1011586. doi: 10.1371/journal.pgen.1011586 (PMC11819509; doi:10.1371/journal.pgen.1011586)
Supplement: Attachment — Submitted filename: response to reviewers_Eldridge-Thomas et al.docx [file pgen.1011586.s021.docx]

Dear Editors and Reviewers,

We are delighted to send our revised manuscript (Ref: PGENETICS-D-24-00494), now entitled, “The transmembrane protein Syndecan is required for stem cell survival and maintenance of their nuclear properties.”

Please find below a detailed list of responses to comments raised by reviewers and the associated changes we have made in the manuscript. We hope you agree with us that the manuscript has improved because of these changes.

Best wishes,

Golnar Kolahgar

**Reviewer #1 (**Evidence, reproducibility and clarity (Required)):

**Summary:** In this paper the authors explore the function of Syndecan in Drosophila stem cells focussing primarily on the intestinal stem cells. They use RNAi knockdown to conclude that Syndecan is required for long term stem cell maintenance as its knockdown results in apoptosis. They suggest that this effect is independent of LINC complex proteins but is associated with changes to nuclear morphology and DNA damage. They go on to show that a similar impact on nuclear shape can be seen in larval neuroblasts but not in stem cells of the female germline.

**Major Comments:** The key conclusion that underpins the paper is that reduced Syndecan causes loss of stem cells. This is based entirely on evidence from cell-type specific RNAi using 3 independent RNAi lines. Overexpression has no phenotype and there is no analysis of loss of function mutants. SdcRNAi3 gives strong phenotypes that are statistically significant and is used throughout the paper. SdcRNAi2 gives comparatively moderate phenotypes which trend in the same direction but it is not clear if these are statistically significant (Fig S1). SdcRNAi line 1 appears to have very little effect (and if anything trends in the opposite direction in S1A). In addition, the knockdown efficiency of the three lines has not been assessed. Another possible concern given the dependence on RNAi3 is that the RNAi control line used is not an ideal match for the VDRC GD RNAi lines as it is in a different genetic background. In order to robustly draw conclusions: the phenotypes with RNAi lines 1 and 2 should be tested for significance; the extent of knockdown in each should be quantified either by qPCR in whole tissue knockdown, or by staining for protein levels if possible, to assess whether the variation in phenotypes is due to different knockdown levels. The use of a loss of function mutant in clones or tissue specific CRISPR-Cas9 KO or KD would also significantly increase confidence in the findings.

- Our qPCR data indicate that Sdc RNAi3 produces the most efficient knockdown, whilst Sdc RNAi1 generates the weakest knockdown. We have incorporated this data in Fig S2A’ (former Fig S1). Knockdown efficacy of Sdc RNAi3 has also been previously reported (e.g. Eveland et al., 2016) and we have added this information in the text.

- We have added the statistical tests on phenotypic categories in Fig S2A and Fig S2B (former figure S1A/B). All Sdc RNAi phenotypic distributions are significantly different to that seen for age-matched controls (p<0.0001 for all).

- We have added in Fig S2D data showing that despite weaker knockdowns with Sdc RNAi1 and Sdc RNAi2, there is still significant ISC depletion after 28 days of RNAi expression, demonstrating the clear role of Sdc in maintaining ISCs.

Similarly, the evidence for a lack of LINC protein role in the phenotype relies on single RNAi lines without validation of knockdowns. The authors should ideally validate these lines in this system or reference other studies that have validated the lines in this or other contexts.

- We have modified the text to add this information (line 220). Moreover, for Msp300 RNAi, we have used a second RNAi line which gave us similar results.

**Minor Comments:** The figures are generally very clear but some of the IF image panels are very small and require significant on-screen enlargement to be legible. In particular in Figure 1B the cross section views make it difficult to assess expression in the different cell types (and don't show very many cells), could this be shown in wholemount or as separated channels in a supplementary figure? In addition, it would strengthen the argument to include counterstains for markers of the different cell types (particularly to distinguish ISC/EB from EE). This could include esg-lacZ to mark ISC/EBs or prospero for EEs. However, if a broader view of these panels makes it clearer that all epithelial cells are expressing Syndecan this may not be essential.

- We have modified Fig 1C to show separated channels and have incorporated larger fields of views with co-immunostaining with different cell type markers in new Fig S1.

Syndecan is referred to throughout as a stem cell regulator. This implies that in certain contexts or in response to certain stimuli its expression may be altered to elicit a stem cell response but no examples of this are shown. Moreover, only knockdown and not overexpression gives phenotypes suggesting its role may be as a required protein than a regulator. Either examples of its expression being modulated in homeostasis or in response to a challenge could be included or the wording could be amended.

- We agree with the reviewer and have amended the wording throughout the text. The title of the manuscript has also been changed from: “The transmembrane protein Syndecan regulates stem cell nuclear properties and cell maintenance” to “The transmembrane protein Syndecan is required for stem cell survival and maintenance of their nuclear properties.”

Expression of Syndecan in neuroblasts is described as data not shown, it would be better to include this for completeness.

- We have added this data in new Fig S7, where we show co-immunostainings with various markers to show the cell types expressing Sdc in the larval brain.

In addition to the intestinal validation of the Syndecan RNAi lines, validation of knockdown in the germline would be valuable to support the conclusions of Fig S4 given differences of knockdown in the germline with some RNAi lines (although inclusion of Dicer in the driver line should have overcome this).

- Sdc expression is very low in the germline, compared to the surrounding somatic cells, therefore we are not confident that we can detect differences in expression level after knockdown. Sdc RNA levels are now added in Fig S6D to show the low expression. We have also added comments in the text (line 266/ line 333).

**Reviewer #1 (Significance (Required)):** The study describes a potentially very interesting, novel link between Syndecan, nuclear shape and apoptosis in cycling cells that could have broad relevance. If fully validated this could have implications for other stem cell populations, including those in mammals and disease relevance in the context of cancer. The paper is fundamentally descriptive in nature and so the level of significance hinges on the strength of evidence and how interesting the phenotype itself is. At this stage the audience will be primarily in the areas of fundamental research in biology of the nucleus and cytoskeleton. Defining the mechanistic link between Syndecan and nuclear morphology will be a critical next step and while not essential for this study would significantly increase the likely interest in the paper.

- We thank the reviewer for these constructive comments. We agree that discovering the mechanistic links between Syndecan and nuclear morphology in future studies, in this and other model systems, will be relevant to many areas of biological research.

In terms of significance in stem cell biology the distinction between a regulator and a requirement to prevent stem cell apoptosis is important and the lack of evidence for a context in which Syndecan plays a regulatory role somewhat detracts from the breadth of impact. My field of expertise is in epithelial stem cell biology.

- We agree and have amended our wording.

**Reviewer #2** (Evidence, reproducibility and clarity (Required)): 
**Summary:** Stem cell (SC) maintenance and proliferation are necessary for tissue morphogenesis and homeostasis. The basement membrane (BM) has been shown to play a key role in regulating stem cell behavior. In this work, the authors unravel a new connection between the receptor for BM components Syndecan (Sdc) and SC behavior, using Drosophila as model system. They show that Sdc is required for intestine stem cell (ISC) maintenance, as Sdc depletion results in their progressive loss. At a cellular level, they also find that Sdc depletion in ISCs affects cell survival, cell and nuclear shape, nuclear lamina and DNA damage. In addition, they show that the defects in shape are not related to cell death. They also find that Sdc depletion in neural stem cells also results in nuclear envelope remodeling during cell division. This is in contrast to what happens in female germline stem cells where Sdc does not seem to be required for their survival or maintenance. 
In general, I believe that this work unravels a connection between Sdc and stem cell behavior. However, I think the study is still at a preliminary stage, as how Sdc regulates different facets of stem cell behavior remains unclear. 

**Major comments:** 
1. To clearly show that the cellular changes produced by loss of Sdc are not due to cell death, one should quantify the ISC area and shape of Sdc-depleted ISCs expressing DIAP1 and compare it to that of Sdc-depleted ISCs. As DIAP1 overexpression only partially rescues ISC loss due to Sdc depletion, one should show that the Sdc-depleted ISCs expressing DIAP1 that still show cellular changes are not dying, as overexpression of Diap1 might not be sufficient to completely rescue cell death in all Sdc-depleted ISCs. In fact, apoptosis in Sdc depleted guts and the ability of Diap1 overexpression to rescue cell death should be analyzed using markers of caspase activity, this will provide a better idea of the contribution of apoptosis to the phenotypes associated to Sdc depletion.

- Our immunostainings with anti-Caspase 3 or Drice do not pick up physiological apoptotic ISCs in the fly gut. This is not entirely unexpected, as apoptosis is unfortunately not easily detected in this tissue. In the absence of a clear positive readout of apoptosis, we cannot discriminate between apoptotic and non-apoptotic stem cells when quantifying area and shape.

- We have however quantified the area and shape of Sdc-depleted ISCs expressing DIAP1 and compared it to that of Sdc-depleted ISCs. This is shown in new Fig S4 and results are explained in the text, lines 170-176 “ISC-specific DIAP1 expression, with or without concomitant Sdc depletion, also resulted in larger cells (Fig S4A). In all genotypes, larger cells were more convoluted (Fig S4B-E), with a stronger effect seen when Sdc RNAi was also expressed (Fig S4C&E). Focussing on these larger (>50μm^2^) cells, we observed similar deformations when Sdc RNAi was expressed both with and without DIAP1 (Fig S4F-G), suggesting that these cells shape changes are not primarily caused by apoptosis.”

2. The authors show that ISC loss is associated with reduced cell density, suggesting that this is most likely due to failure in new cell production. What do they mean with cell production? Is this related to a problem in regulating cell division or to the fact that as some ISCs are lost by apoptosis there is progressively less ISCs or to a combination of both? I think that cell division should be monitored throughout time as well as cell death in ISCs.

- Based on esgF/O experiments (Fig. 1D-F and S2C) where we can trace the production of new cells with GFP, we know that Sdc RNAi expression (i) impairs the appearance of newly differentiated cells in the tissue and (ii) results in the disappearance of progenitor cells (Fig. S2C). Supporting these points, (i) we have observed PH3+ mitotic stem cells upon Sdc RNAi, so we are confident the cells are able to initiate cell division (see also Fig. 2G), and (ii) we have occasionally noted in fixed samples stem cells looking like they were in the process of delaminating. Overall, the failure of cell production is likely related to problems with both completion of cell division and progressive stem cell loss. High resolution live imaging will in future give us a better insight into stem cell division dynamics/behaviour, however, the technical improvements required are beyond the scope of this project. In the meantime, we have clarified our statement in the text (lines 114-117).

3. The authors report that in contrast to what happens when Sdc is eliminated from ISCs, its elimination from EEs results in an increase in the number of these cells. An explanation for this result is missing.

- We have amended the text, lines 137-139 “Based on known roles of Sdc in other *Drosophila* tissues [22–25], this could be due to Sdc modulating Slit/Robo signalling involved in EE specification [26,27].”

4. The authors suggest that "Sdc function is unlikely to be fully accounted for by individual LINC complex proteins, although these proteins might act redundantly". Checking redundancy seems a straight forward experiment, which only requires the simultaneous expression of RNAis against several of these proteins. This would help to settle the implication of LINC complex proteins on Sdc function. 

- To check for redundancy, we have combined Klaroid RNAi with Msp300 or Klarsicht RNAis and stained the tissues for H2Av. We did not detect the same levels of ISC DNA damage as with Sdc depletion (see new panel C, in Fig S5). The text is amended (lines 232-235).

5. Although quantification of DNA damage, by immunolabelling with γH2Av, reveals that knockdown of individual LINC complex components did not recapitulate the damage observed upon Sdc depletion (Fig.3G), the image shown in Fig.3F reflects much higher levels of γH2Av in Msp300 RNAi cells compared to Sdc RNAi cells. Authors should clarify this.

- Like the reviewer, we are intrigued by the higher levels of H2Av staining in the tissue, despite Msp300 knockdown in stem cells only (Fig 3F). It is worth noting that we observed this with two independent RNAi lines (we showed only one RNAi in the manuscript). In fig. 3F, we have indicated with an arrow the only ISC that is H2Av positive, and mention in the text that the majority of DNA damage signal observed in the Msp300 RNAi condition is in enterocytes, not ISCs (amended text lines 237-239). We currently do not have an explanation for why loss of Msp300 in ISCs should cause DNA damage in neighboring cells.

In addition, the consequences of the simultaneous elimination of more than one component of the LINC complex on DNA damage should be analyzed.

- We agree and have checked for redundancy (as in point 4).

6. The authors claim that the fact that "DNA damage was found more frequently in Sdc-depleted ISCs with lamina invaginations compared to those without (Figure 3H), supports a model whereby the development of nuclear lamina invaginations precedes the acquisition of DNA damage". However, to me, these results show that there is a relation between these two phenotypes, but not that one precedes the other. In order to show which one is the possible cause and which the consequence, the authors should perform a time course of the appearance of each of these phenotypes.

- We agree with the reviewer. We have rephrased our statement to indicate a relationship between lamina invaginations and DNA damage, rather than a causality. See lines 243-247: “We carefully examined the frequency of both nuclear lamina invaginations and DNA damage in a large number of ISCs (Fig 3H). DNA damage was found more frequently in Sdc-depleted ISCs with lamina invaginations compared to those without (Fig 3H), demonstrating a relationship between acquisition of DNA damage and disruption of the nuclear lamina.”

- In terms of performing a time course analysis, the difficulty is that after 3 days of Sdc RNAi expression, the apparent DNA damage (Fig 3G) corresponds to a very small proportion of stem cells, meaning that an exceptionally large sample size would be required to achieve robust statistical analysis.

7. When studying the role of Sdc in neural stem cells, the authors show that elimination of Sdc in neuroblasts also affect nuclear envelope and shape. Furthermore, in this case, they also show that Sdc elimination affects cell division. To look for a more conserved role of Sdc in stem cell behavior, I believe the authors should also analyze whether Sdc elimination in neural stem cells results in an increase in DNA damage, as it is the case in ISCs.

- We have stained larval brains for H2Av to see if DNA damage is also observed following Sdc knockdown in neuroblasts. The results are shown in new Fig S8A-C and new text (line 282) has been added: “These defects did not elicit detectable induction of DNA damage (Fig S8A-C).”

8. When analyzing a possible role of Sdc in fGSCs, quantification of germline stem cells and γH2Av levels in control nosGal4 and nos>Sdc RNAi germaria should be done. In addition, it is not clear to me whether Sdc is in fact expressed in fGSCs.

- As mentioned in comments to reviewer 1, we have added a panel (Fig S6D) to show the low Sdc expression in fGSCs and have amended the text.

9. The authors should show presence of Sdc in neuroblasts.

- Yes, we agree, as also mentioned in comments to reviewer 1.

**Reviewer #2 (Significance (Required)):** In general, although this work reveals that elimination of Sdc affects different aspects of intestinal and neural stem cell behavior, including cell survival, cell production, nuclear shape, nuclear lamina or DNA damage, their contribution to stem cell loss and interactions between them have not been analyzed in detail. The role of the basement membrane in stem cell behavior has been extensively studied. In particular, the role of syndecan in stem cell regulation has been primarily confined to cancer, muscle, neural and hematopoietic stem cells. Thus, the study here presented could extend the role of Sdc to intestinal stem cells and could potentially reveals a conserved role for Sdc in neural stem cell behavior. However, the problem with the data mentioned above, hinders the assessment of the significance of this work. 

- We thank the reviewer for their assessment and are glad that they also find that our study provides novel connections between Syndecan and the regulation of intestinal and neural stem cell behaviors. To strengthen our conclusions, we have included additional experiments and amended the text, as indicated above.

**Reviewer #3** (Evidence, reproducibility and clarity (Required)): 
Peer-review: The transmembrane protein Syndecan regulates stem cell nuclear properties and cell maintenance. 

In this work, the authors investigate the role of the transmembrane protein Syndecan (Sdc) in nuclear organisation and stem cell maintenance. Theys show that Sdc knockdown in intestinal stem cells (ISCs) results in a reduction of the ISC pool as well as of their progeny. They hypothesise that these ISCs might get eliminated via cell death, however, expression of the apoptotic inhibitor DIAP1 only rescued ISC loss by 50%. Hence, they suggest that apoptosis can not account for the total decrease in ISCs observed upon Sdc loss. 
ISCs depleted from Sdc exhibited abnormal cytoplasmic and nuclear morphologies. As Sdc has previously been implicated in the abscission machinery in mammalian cultured cells, they tested if Sdc could be playing a similar role in the abscission of ISCs. However, ISCs were capable of undergoing cytokinesis. Next, they tested if Sdc depletion could be altering the linkage between the plasma membrane and the nucleus mediated by the Linker of Nucleoskeleton and Cytoskeleton (LINC) complex. However, individual knockdowns of the different components of the complex did not disrupt the nuclear morphology to the same extent as Sdc knockdown, suggesting that Sdc function may be independent of the LINC complex. Finally, they observed that Sdc-depleted ISCs exhibited DNA damage, suggesting that Sdc may play a role in DNA protection. 
The authors next tested if Sdc played similar roles in other stem cell types such as the female germline stem cells (fGSCs) and larval neural stem cells (NSCs). While Sdc depletion appeared dispensable for fGSC maintenance, it prolonged NSC divisions and altered the nuclear morphology of NSCs. Upon further investigations, they observed that the NSC's nuclear envelope was disrupted upon division, hence causing defects in the nuclear size ratio of NSC and their progeny. 
This study provides with interesting findings in the field and proves a new role for Sdc in the regulation of intestinal and neural stem cell maintenance. I would recommend this manuscript to be accepted if the authors address the following comments. 

**Major comments:** 
1. In Figure 2 A-B, Sdc RNAi should ideally have a UAS control transgene to match the number of UAS being expressed to that of Sdc RNAi, DIAP1. Otherwise, it is plausible that reduced RNAi expression of Sdc RNAi, DIAP1 animals is the cause of the partial rescue. Staining against cell death markers such as Dcp-1 or TUNEL might also quantify the number of cells undergoing cell death in each of the genotypes.

- As mentioned in comments to reviewer 2 (point 1), it is difficult to label apoptotic ISCs in the fly gut.

2. " These phenotypes were observed both with and without DIAP1 expression (Figure 2C), indicating that these cell shapes are not caused by apoptosis." Misleading, as DIAP overexpression in Sdc knockdown background only rescued apoptosis by 50%. Hence, it is possible that those cells undergoing morphological defects, protrusions and blebbing might still undergo death - also considering those morphological changes are typically observed in apoptotic cells... Therefore, to rule apoptosis out, these cells should be shown to be negative for cell death markers.

- We agree, however, it is difficult to label apoptotic ISCs. We think that the quantification shown in new Fig S4 support the notion that the cell shapes induced by Sdc depletion are not primarily caused by apoptosis (see also comments reviewer 2, point 1).

3. Show if Sdc is expressed in fGSCs - the lack of phenotype caused by Sdc knockdown might be due to lack of expression of Sdc.

- As mentioned in comments to reviewers 1&2, we have added data showing the low *Sdc* expression in fGSCs (Fig S6D).

4. "After confirming the presence of Sdc in neuroblasts (data not shown)." Data should be shown. It would be of great interest for researchers if you showed a staining of different brain cell types (NBs, glia, neurons) and the Sdc expression patterns.

- As mentioned in comments to reviewers 1&2, we have added this information in new Fig S7.

5. You show how Sdc-depleted NBs have disrupted nuclear morphologies. However, does Sdc KD in NB lineages affect their ability to self-renew and generate differentiated progeny? Is the number of NBs and of their progeny cells altered as it is for ISCs?

We have knocked down Sdc in NBs and quantified brain lobe size in 3^rd^ instar larvae (new Fig S8D). We have not observed deleterious effects on size, and commented in the text, lines 299-301 “brain lobe sizes from larvae with Sdc-depleted neuroblasts were comparable to control brains (Fig S8D) suggesting that Sdc depletion does not affect neuroblast ability to generate differentiated progeny.”

6. Does protection against DNA damage in an Sdc knockdown background prevent the defects observed with the single knockdown and ISC elimination?

- This is a good question, and we have emphasized this point in the discussion (line 343). However, because of the multiple routes of DNA damage response, and the multiple lines needed to explore this connection, we feel that investigating this question is beyond the scope of this project.

7. Any idea the similarities between ISC and NBs that can account for why Sdc knockdown has effects in those systems, while no effect was observed in the germ cells?

- Besides the differences in expression level, we speculate that fGSCs may have a different nuclear / lamina architecture which might reflect differences in how GSCs control the physical integrity of their nuclei. It is also possible that the differences observed between tissues reflect the way stem cells connect to their microenvironment. Notably, fGSCs rely extensively on E-Cadherin mediated adhesion with neighbouring cells, and it is possible that contact with the extracellular matrix is dispensable. We have considered these possibilities in the discussion, lines 331-340.

**Minor comments:** 
8. Lamina invaginations, for example in Figure 3 A, could be indicated with an arrow for easier detection.

- Thanks for this suggestion, we have added yellow arrowheads in the figure.

9. Specify the type and location of NB imaged during live cell experiments.

- The NBs were imaged in the brain lobes, and we did not distinguish between type I and II NBs. We have added a sentence in the method section to clarify (lines 556-557).

Reviewer #3 (Significance (Required)): 
Expertise: Drosophila stem cells
