# Supplementary material for: The transmembrane protein Syndecan is required for stem cell survival and maintenance of their nuclear properties
Source: PLoS Genet. 2025 Feb 6;21(2):e1011586. doi: 10.1371/journal.pgen.1011586 (PMC11819509; doi:10.1371/journal.pgen.1011586)
Supplement: Attachment — Submitted filename: Response to reviewers_Eldridge-Thomas et al_Jan2025.docx [file pgen.1011586.s022.docx]

Response to reviewers: (14^th^ January 2025)

Dear Professor Bosco,

Thank you for sharing the additional feedback on our manuscript “The transmembrane protein Syndecan is required for stem cell survival and maintenance of their nuclear properties”. We were pleased to read that reviewer 1 was satisfied with the additional data provided. Our responses to the remaining points raised by Reviewer 2 are shown in blue.

*ReviewersReviewer #1: The authors have addressed most of my concerns.*

*Reviewer #2: Summary of Revisions:

In this revised manuscript the authors have addressed most of the issues raised but a few points remain.

The paper relies on data from RNAi knockdown of Sdc with the main figures using a single RNAi line and two additional lines used for validation in the supplementary figures. The authors have now added the requested statistical analysis to FigS2A confirming the significance of the knockdown effects on cell production for all three lines. However, it should be noted that at the 14-day timepoint Line 1 appears to show a significant difference in the opposite direction to the other two (increasing production of new differentiated cells) which should be reflected in the text. At 28 days the trend is the same direction (albeit far weaker for line 1), but Lines 2 and 3 are very consistent, which allays concerns about inconsistencies between lines.

The authors have also now included qPCR validation of the knockdowns showing knockdowns of around 50, 60 and 70% for lines 1, 2 and 3 respectively. However, this is based on n=1 replicate making it difficult to draw conclusions about the relative knockdown efficiencies. This data should be at least triplicated. In addition, Sdc is enriched in ISC and EBs and the quantification is performed after 14 days of knockdown, by which point the proportions of ISCs and EBs have changed significantly between lines. It is therefore unclear to what extent this represents differences in knockdown efficiency as opposed to loss of the cell types that express Sdc. Analysis of knockdown at an earlier timepoint may help address this.

While it is not implausible that there may be a critical threshold of knockdown a range of 50-70% is given as sufficient difference to explain the large difference in strength of phenotype. This may be relevant to the point raised by reviewer 3 regarding UAS dose compensation in the rescue experiments with Diap1 (Fig 2) as it suggests a relatively small suppression of SDC RNAi expression and therefore Sdc knockdown due to an extra UAS copy driving expression of DIAP1 could be sufficient to impact the phenotype and appear as a rescue.

The inclusion of knockout data either from mutant clones or cell-type specific CRISPR would significantly increase confidence in the findings.

LINC protein knockdowns. The authors have included references to confirm the validation of these lines in other studies supporting their use here.

Figure clarity. The authors have modified Fig 1C as requested to split channels and included additional markers in S1. The additional data is very helpful in clarifying the expression patterns.

Sdc as regulator. The text has been amended to reflect that Sdc “is required” rather than acting as a regulator addressing this point. This does somewhat detract from the potential breadth of impact of the findings as a regulatory role may have broader implications than a requirement.

Expression of Sdc in neuroblasts. Authors have included the requested data.

Knockdown validation in germline. The authors have included additional data to show very low-level expression of sdc RNA in the germline stem cells. It is not totally clear from the data presented that Sdc protein is expressed in the GSCs but the conclusion that it is not required there would still stand.*

 *Remaining Points:*

*1. Phenotype direction of Sdc RNAi Line 1 in S2A should be acknowledged in the text.*

In Fig S2, we show representative images of the phenotypes observed upon expression of different Sdc RNAi lines for 14 or 28 days in progenitor cells and their subsequent progeny. The associated quantifications show that expression of RNAi line 1 initially increases the production of new differentiated GFP-positive cells, whereas the other lines, at this time point, start showing a reduction in small progenitor cells. We have amended the text to reflect this: line 114, ‘expression of two out of three independent RNAi lines impaired new cell production […]’, and in the Fig S2 legend, line 1043, ‘however, in (A) Sdc RNAi 1 shows significance in the opposite phenotypic direction to Sdc RNAi 2 & 3.’

*2. Knockdown quantification for the three Sdc RNAi lines should be replicated and may be better assessed at an earlier timepoint.*

To better quantify Sdc RNAi-mediated knockdown, we have expressed each RNAi line with a ubiquitous driver (*tub-GAL4, UAS-mCD8-GFP*) and extracted the total RNA from whole third instar larvae. Our measurements indicate two out of three lines efficiently reduce overall Sdc transcripts by more than 80%, while RNAi line 1 does not produce a strong knockdown. The data is shown in Fig S2A’.

*3. Knockout data would strengthen the findings of RNAi experiments.*

We agree with the reviewer and have highlighted in the discussion the need for future tools to address which parts of the protein are required (lines 385, 386). In the meantime, I am afraid this is beyond what we can achieve with these revisions. Yet, we believe our cell-specific knockdown results are useful at this stage. In the meantime, because we have observed similar phenotypes with two strong RNAi lines, we feel confident about our findings.

*4. Rescue experiments involving DIAP1 expression require UAS-dosage balance controls.*

While we can understand Reviewer 2's concern, the point we are making in the revised manuscript is that the primary cellular deformations caused by Sdc knockdown are unlikely to be caused by apoptosis. On page 7 (line 176), we had added ‘we observed similar deformations when Sdc RNAi was expressed both with and without DIAP1 (Fig S4F-G), suggesting that these cells shape changes are not primarily caused by apoptosis.’ Potential issues with UAS-dosage would have presumably led to a reduction in cellular deformations, which we did not see.

We hope you find our amendments satisfying, and that our manuscript fully meets PLOS Genetics's publication criteria.

Best wishes,

Golnar Kolahgar
